# Displacement Estimation Based on Optical and Inertial Sensor Fusion

**DOI:** 10.3390/s21041390

**Published:** 2021-02-17

**Authors:** Tomasz Ursel, Michał Olinski

**Affiliations:** Faculty of Mechanical Engineering, Department of Fundamentals of Machine Design and Mechatronic Systems K61W10D07, Wroclaw University of Science and Technology, Łukasiewicza St. 7/9, 50-371 Wroclaw, Poland; michal.olinski@pwr.edu.pl

**Keywords:** stationary state, zero velocity, ZV, accelerometer, optical-flow sensor, OFS, symmetrization, linearization, complementary filter, double integration

## Abstract

This article aims to develop a system capable of estimating the displacement of a moving object with the usage of a relatively cheap and easy to apply sensors. There is a growing need for such systems, not only for robots, but also, for instance, pedestrian navigation. In this paper, the theory for this idea, including data postprocessing algorithms for a MEMS accelerometer and an optical flow sensor (OFS), as well as the developed complementary filter applied for sensor fusion, are presented. In addition, a vital part of the accelerometer’s algorithm, the zero velocity states detection, is implemented. It is based on analysis of the acceleration’s signal and further application of acceleration symmetrization, greatly improving the obtained displacement. A test stand with a linear guide and motor enabling imposing a specified linear motion is built. The results of both sensors’ testing suggest that the displacement estimated by each of them is highly correct. Fusion of the sensors’ data gives even better outcomes, especially in cases with external disturbance of OFS. The comparative evaluation of estimated linear displacements, in each case related to encoder data, confirms the algorithms’ operation correctness and proves the chosen sensors’ usefulness in the development of a linear displacement measuring system.

## 1. Introduction

Indoor and outdoor positioning and displacement estimation constitute an important aspect of research nowadays concerning many engineering applications, such as robots navigation, earthquake engineering, as well as systems for health monitoring, elderly care, gait measurement, and pedestrian navigation. In each application, the focus is placed on identifying the angular orientation, such as the roll, pitch, and yaw angles, and/or on the X, Y, and Z linear displacements [1,2].

One of the commonly used methods for the displacement estimation of objects, such as a computer mouse, and robots is the application of an optical flow sensor (OFS). The optical flow is defined as the velocity of relative motion between the observer and adjacent objects such as surfaces, edges, or contrasting points, in consecutive observational frames [3,4]. In [5], a developed flow computing system based on FPGA was used for displacement estimation, but difficulties arose in differentiating between rotation and lateral translation of the robot. In order to minimize the possible errors, two OFS were applied for robot navigation in [6] and in [7]. Eight such computer mice sensors were combined and used for translation and rotation estimation in a ground robot’s odometry. Other examples of OFS usage for visual odometry in that case of ground robots include, for instance, [8,9]. Furthermore, in [10], OFS was studied for outdoor odometry calculation possibilities and a velocity first order correction algorithm, based on the linearization of velocity dependance, was developed. In the case of [11] the OFS was supplemented by an afocal system to compensate for the possible error of displacement estimation caused by the sensor’s change in vertical height over the ground. In [12] the same idea as in [11] was applied with another sensor, and a gyroscope was used, but not as additional tool in distance estimation, but rather to obtain the orientation data. So, the idea in [12] was to improve the operation of OFS itself, while in the case of this article’s research, the aim is to supplement OFS usage with another sensor serving as the second source of displacement data. This is similar to [13], where mobile robot odometry was calculated with the usage of OFS for displacement estimation. Sensor fusion was also applied in this article, but only for the well-known case of angular orientation obtained from an additional inertial measurement unit (IMU) sensor, as in [14]. In addition, OFS-based sensors are even offered by companies like RoboteQ [15] for acquiring a mobile robot’s X-Y localization and yaw angle orientation.

Various other sensors and methods are applied for displacement estimation including not only OFS, but also inertial navigation systems (INS) using sensors such as accelerometers, gyroscopes, and IMU sensors [16,17], as well as magnetometers [18] or EMG signals [19]. For low-cost and simplicity reasons, the application of only inertial sensors is often intended, such as in [20], where the usage of sensors other than an accelerometer and a gyroscope was avoided, and unaided inertial positioning was achieved. Moreover, in comparison to other navigation systems based on GPS, magnetometers or even optical sensors [21], INS have a greater resistance to external interferences, since the gravitational field is a much more accurate reference. Therefore, INS may function in environments such as a building’s interior, underwater, or underground, where GPS signal is restricted. The external influences are also limited, since inertial sensors do not need antennas or openings in their casings.

Furthermore, there are navigation systems designed specifically for gait estimation/pedestrian navigation and, in consequence, focus on displacement estimation, often with the usage of inertial sensors [22]. In addition, human gait is a repeatable activity and provides the opportunity to detect individual states of gait. As a result, a sensor’s zero velocity/acceleration can be associated with the detected still phase, which allows one to implement drift/error corrections [17,23]. Specifically, this is the idea behind the zero-velocity update (ZUPT), which assumes resetting the velocity in stationary states [16]. However, such methods cannot be easily applied in a general case of distance estimation when a non-regular movement occurs. For such situations, the zero velocity (ZV) state detection algorithm may be also determined on the basis of, for example, acoustic noise accompanying the motion. It is an essential factor providing decent information about an object’s movement [24].

The above examples suggest that, in addition to the kind of applied sensor, an equally important aspect of displacement estimation is computations and applied algorithms. For instance, a FIR-filter type displacement estimation algorithm eliminating low frequency drifts is presented in [25]. In another example [26], the application of neural networks and a virtual IMU sensor based on machine learning and the human leg kinematic model is shown. The applied algorithms are essential in the case of an accelerometer, where detecting the zero-velocity (ZV) state (stationary state/still phase) is often required as the reference for the mentioned ZUPT algorithm. The signal processing procedure with the measurements from gyroscopes or an accelerometer is used in most methods of ZV identification, for instance in the local acceleration standard deviation-based approach in [16], where a still-phase or swing phase is detected when this parameter is below the defined level. An adaptive value of such a threshold limiting the ZV detection is applied in [21] for multi-sensor fusion between the accelerometer, gyroscope, and pressure sensor. In [27], for the estimation of stride length and orientation, a ZUPT algorithm with a complementary Kalman filter was used. Again, ZUPT was used in [16] to assist the foot-mounted INS in the extended Kalman filter (EKF) system called INS-EKF-ZUPT (IEZ) to reduce the accumulated accelerometer error and, in consequence, the accumulated velocity error. Even further research resulted in an idea of applying fast Fourier transform (FFT) in various parts of the displacement estimation algorithm. For instance, in [18], a foot–mounted IMU positioning algorithm based on magnetic constraint was supplemented with FFT for improving the distinguishing degree of measured features. In [28], walking or stationary state was detected by using FFT on angular velocities measured by a smartphone. A similar goal, step-detection and step-length estimation, was achieved in [29] with the acceleration signal smoothed by FFT.

The mentioned cases of hardware usage and methods applied are close to this article’s subject of proposing a displacement estimation algorithm. However, our research focuses on displacement estimation in a general case, when the movement is not a repeatable gait, but a motion with unpredictable characteristics. The main aim is to propose a novel sensor fusion of both OFS and accelerometer with usage of a developed complementary filter in order to further improve the estimated displacement, especially in cases when OFS encounters external disturbances. The intention is to enhance the stability of readings and obtain displacement estimation with precision of ±10 cm at a distance of 10 m. The usage of a complementary filter should provide a correct accelerometer OFS measurement system of velocity with minimum 95% certainty at 0.1 m/s deviation and ensure mutual elimination of errors from two sensors. Improving the accelerometer’s existing algorithm for zero-velocity states identification and velocity correction is another new important aspect researched in this article. The basic preliminary version of the accelerometers data processing algorithm was described in our previous work [24,30]. Specifically, the accelerometer’s measured signals are processed according to an algorithm, where ZV detection is achieved through acceleration and velocity data observations and, in addition, the velocity correction is further supplemented by applying acceleration symmetrization (linearization), improving the obtained results. At the end of the paper, the results of displacement estimations obtained for the accelerometer, OFS, and their fusion are presented and compared. The main findings demonstrate that zero-velocity detection could be achieved accurately with the developed accelerometer data processing algorithm. Secondly, the use of sensor fusion significantly improved the stability of the displacement estimation method based on the readings from the accelerometer and OFS. Moreover, the applied simple complementary filter allowed us to detect sensor errors and eliminate them.

## 2. Materials and Methods

### 2.1. Designed Test Stand and Studied Sensors

**Figure 1:**
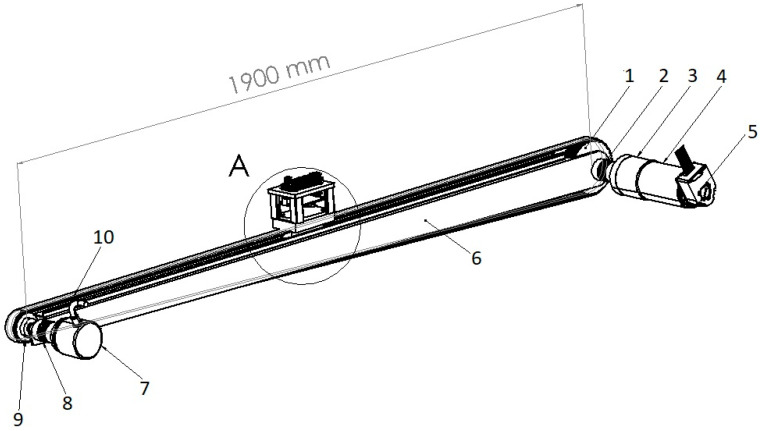
Project of the test stand with linear guide and servo actuator [30].

**Figure 2:**
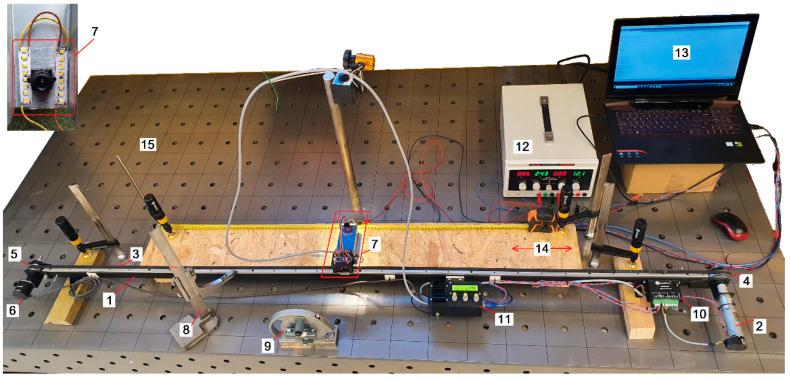
Built test stand for sensors linear displacement measurements.

In order to perform experiments with an accelerometer and optical flow sensor (OFS), it was necessary to design a test stand, as shown in Figure 1, which enabled acquiring linear movement corresponding to a specified motion equation. The final test stand presented in Figure 2 is assembled with: a linear guide (1), a servo drive with a planetary gear (2), a toothed belt HTD 3M (3), a drive wheel (4), a measuring wheel (5), a measuring encoder (6), a measurement platform placed on a sensor suspension with vibration damping system (7), a control system (8), a precise machine level with 0.05 mm/m accuracy (9), the servo amplifier Leadshine DCS303 (10), a kinematic gain controller (11), a power supply (12), a laptop (13), a measuring track (14), and a steady table weighing 850kg (15).

The studied sensors are placed on a platform attached to the toothed belt moved by the drive wheel. A controlled and repeatable displacement can be imposed on the test stand. The obtained translational movement, provided by the used drive and gear, reach a max. speed of 1 m/s, while the average acceleration is at maximum 6 m/s^2^. The location of the studied sensors can be calculated with a resolution of 0.012 mm by utilizing an encoder with 720 pulse/rate resolution. Primarily, Matlab software was used to analyze the data, but eventually the developed algorithm code was implemented in the SAM3X8E microcontroller on the Arduino Due board.

**Figure 3:**
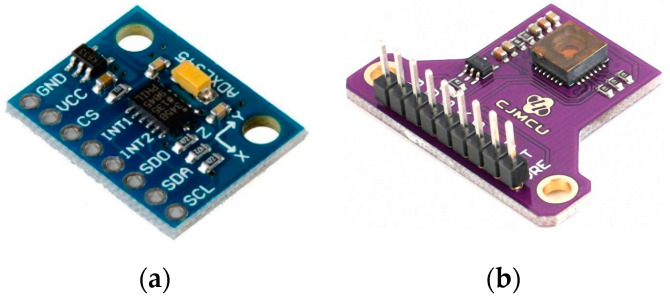
Used sensors: (**a**) ADXL345 accelerometer [33]; (**b**) PMW3901 optical flow sensor [34].

In order to determine the algorithm’s performance, efficiency comparative studies were performed on 2 sensors, the ADXL345 accelerometer (Figure 3a) [31] and the PMW3901 optical flow sensor (OFS) (Figure 3b) [32]. The tested OFS communicates via SPI, while the accelerometer has an analog output. Throughout the tests and final experiment, the platform produced acoustic noise while moving on a fairly flat surface. This smoothness allowed us to assume that the platform’s orientation remains unchanged, with the exception of the yaw angle. However, since this research concentrated on linear displacement, this angle was stabilized and the need to refresh it was omitted. It was assumed that the tolerance for the ground flatness deviation did not exceed 1 cm over an area of 25m^2^. The distance to the ground was constant. Light was dosed by artificial lighting and the measuring area for the optical sensor was isolated from the influence of external light.

The following subsections present the algorithms applied for measured data postprocessing in the case of the studied accelerometer and OFS. Finally, a method of both sensor fusion with usage of a complementary filter was developed, and this is introduced in Section 2.4.

### 2.2. Accelerometer—Displacement Estimation Algorithm

**Figure 4:**
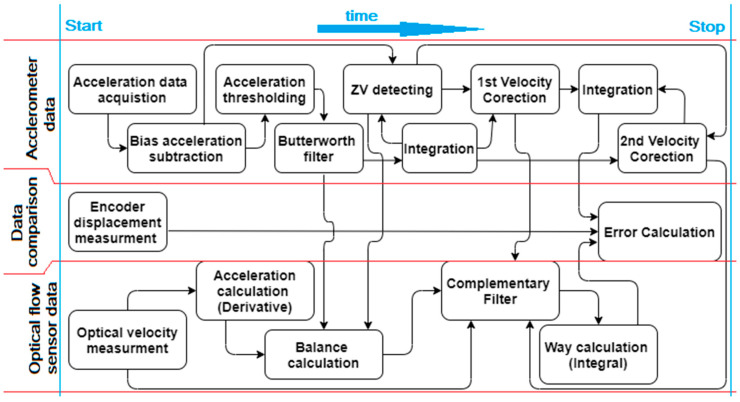
The flowchart of data processing algorithm for displacement (way) estimation concerning the accelerometer, optical flow sensor (OFS), complementary filter, and error calculations in relation to the encoder.

**Figure 5:**
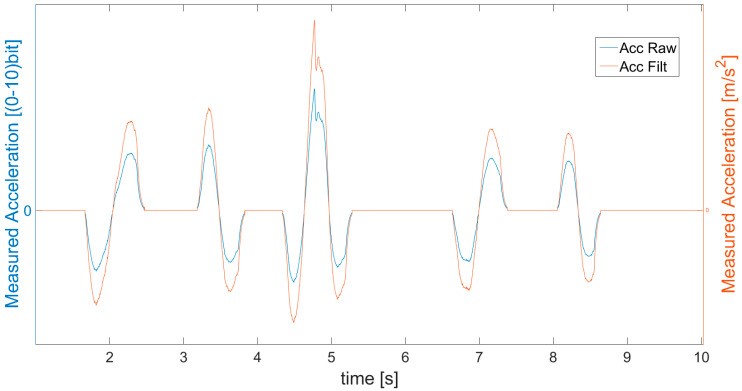
Measured acceleration (blue line) and data after bias subtraction, saturation, scaling and filtration (red line—*a_SCAL_ACC_*).

The overall algorithm is shown in Figure 4, including procedures for the accelerometer, OFS, sensor fusion and results evaluation in the form of error calculations. The algorithm’s operations and steps are explained below, as supported by the equations and figures. The first step is acceleration data acquisition, with the usage of an accelerometer supplemented with an analog filter to remove spikes. These data are presented in Figure 5 as a blue line and are indicated as a_raw_ACC_. In order to be usable in real applications, the acquired data had to be further properly filtered.

The accelerometer measured the acceleration in a local coordinate system in 3 axes. The orientation of this coordinate system in accordance to the Earth gravitational field was not known, but it created a bias of measurements in all 3 axes, which had to be compensated. It was performed by measuring the accelerometer’s indications, in a stationary state, in each axis for about 1000 samples and calculating their average. Subtracting these initial values from each axis eliminated the influence of gravity. In the case of the presented algorithm and experiments, only the linear acceleration in the direction of movement is important, so the other two axes were omitted and all considerations concerned only measurements of this axis. Acceleration values after bias compensation are indicated in equations as the *a_LIN_*__*ACC*_. Furthermore, in order to remove the measuring noise, saturation of the obtained data is applied to obtained *a_SAT_*__*ACC*_, following Equation (1) expressed as:

(1) $$a_{SAT\_ACC\;n}=\{\begin{array}{c} 0\\ a_{LIN\_ACC\;n-1}\\ a_{LIN\_ACC\;n} \end{array}\begin{array}{c} for\;a_{LIN\_ACC}<a_{SAT\_MIN}\\ for\;\left(a_{LIN\_ACC\;n-1}-a_{LIN\_ACC\;n}\right)\geq a_{SAT\_MAX}\\ for\;other\;cases \end{array}$$

It means that too small values of acceleration (lower than assumed value of *a_SAT_*__*MIN*_) are neglected and treated as 0. In addition, the measurement errors of too fast increases in values reaching at least the *a_SAT_*__*MAX*_ are corrected by checking the derivative. The parameters such as *a_SAT_*__*MIN*_, *a_SAT_*__*MAX*_, and others in the following parts of the article have constant values and are defined experimentally (by analyzing graphs), when tuning of the filter is performed. If an error is detected, the incorrect value is replaced with the previous one. After that, the values of acceleration are scaled from bit form to m/s^2^, indicated as *a_SCAL_*__*ACC*_, filtered with Butterworth filter, and presented as a red line in Figure 5 (Acc Filt). The filtering operations of bias compensation, saturation, and scaling constitute the second, third, and fourth steps of the algorithm indicated in Figure 4.

**Figure 6:**
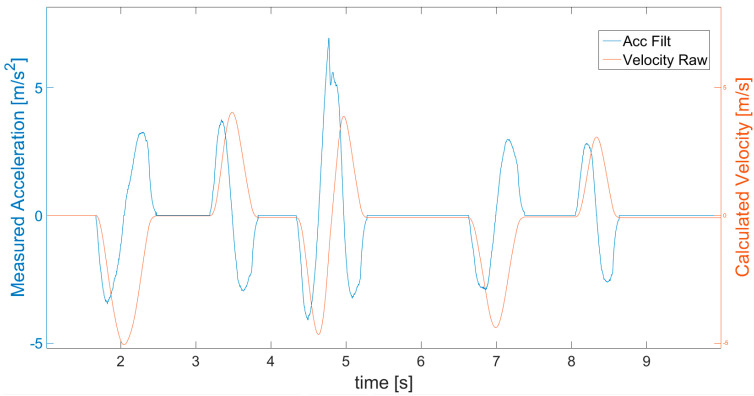
Velocity (red line) calculated with simple derivative of filtered acceleration (blue line).

The first calculation of velocity *V_I_* (Figure 6) is performed, by simple integration of filtered acceleration values, according to Equation (2) expressed as:

(2) $$V_{I\;n}=\sum_{n=0}^{n}a_{SCAL\_ACC\;n}*dt$$

**Figure 7:**
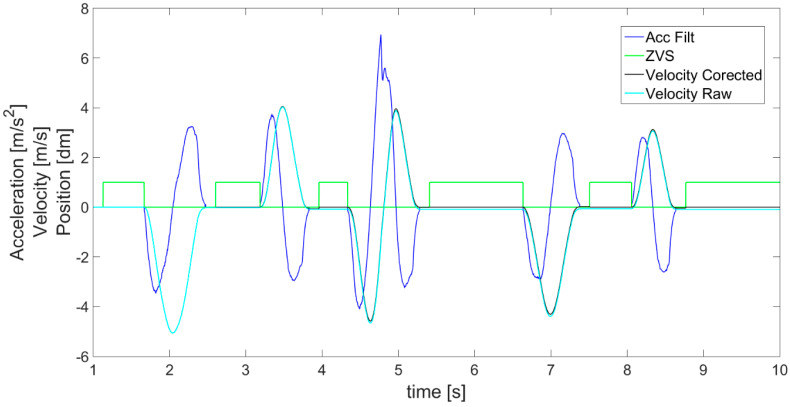
Detection of zero velocity states (ZVS indicated as green line) and 1st velocity correction.

The obtained velocity *V_I_* is used together with acceleration *a_LIN_*__*ACC*_ in the next step of the algorithm, which is zero velocity state (*ZVS*) detection (Figure 4). Below, the parameter *ZVS* is introduced in order to indicate the detection of zero velocity state (*ZVS* = 1) defined by Equation (3) as follows:

(3) $$\{\begin{array}{c} ZVS=1\\ ZVS=0 \end{array}\begin{array}{c} for\\ for \end{array}\begin{array}{c} \left(T_{a}\left(a_{LIN\_ACC}\leq a_{Noise}\right)\geq T_{MIN}\right)\wedge \left(V_{I}\leq V_{MIN}\right)\\ a_{LIN\_ACC}\geq a_{Noise} \end{array}$$

where *T_a_* denotes the detected period of time in which acceleration is lower than the assumed level of *a_Noise_*. Specifically, the ZVS is detected when the obtained value of earlier acceleration *a_LIN_*__*ACC*_ is close to 0 for an identified experimentally minimum length of time period *T_MIN_*. This introduces a delay, but is necessary to avoid *ZVS* detection when, for instance, acceleration quickly passes the 0 value as it changes its sign. For this reason, in Figure 7, it is visible that the *ZVS* (green) detection starts with a delay after the acceleration (blue) decreases to 0. In fact, a moving object never has a velocity so perfectly constant that the accelerometer would not detect some changes. The object always accelerates or decelerates a little. In addition, to avoid detection of *ZVS* in cases of experiencing approximately uniform motion (without acceleration), the values of velocity *V_I_* (Figure 7 cyan) are checked for being below the assumed level *V_MIN_* (close to 0). This way, the *ZVS* detection according to acceleration is supplemented by the search for movement periods according to velocity. Generally, *ZVS* is detected only when both conditions for low values of acceleration and velocity are met for a determined period of time, and therefore in cases of high enough values of velocity (larger than *V_MIN_*), or non 0 acceleration (larger than *a_NOISE_*), it is impossible to distinguish the *ZVS*.

**Figure 8:**
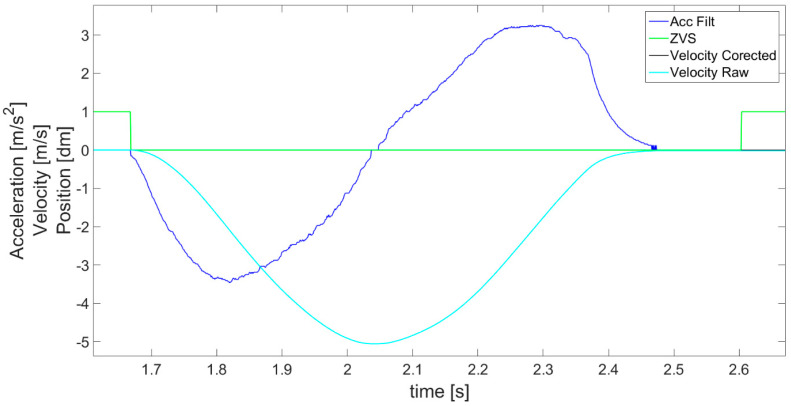
Magnification of part of Figure 7 presenting the effect of the first velocity correction based on coefficient subtract in the detected ZVS periods.

According to *ZVS* detected in Figure 7, the first velocity correction is performed, following Equation (4), by subtracting a velocity correction coefficient *V_COR_* equal to the velocity measured from time to time in detected *ZVS*. This way, the velocity value is brought to 0 in the stationary states. The results of such corrected velocity are presented in Figure 7 and Figure 8 as a black line compared with the uncorrected velocity *V_I_* as a cyan line. A fault is visible at the beginning of the detected *ZVS* area, for instance at about the 4th second in Figure 7.

(4) $$V_{I\_COR}\left(V_{I},ZVS\right)=\{\begin{array}{c} 0\\ V_{I}-V_{COR} \end{array}\begin{array}{c} for\;ZVS=1\\ for\;ZVS=0 \end{array}$$

**Figure 9:**
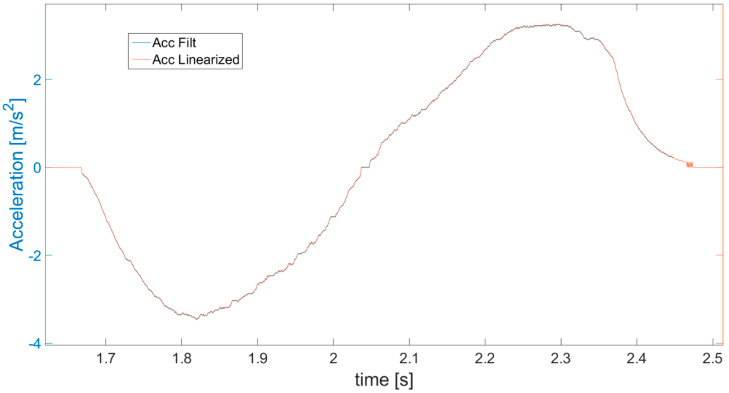
Acceleration values calculated after performing the symmetrization (Acc linearized).

After the above operations, the algorithm’s key 2nd velocity correction is performed (Figure 4). The detected idle periods (ZVS in Figure 7) are first used to perform symmetrization (linearization) of acceleration a_SCAL_ACC_ from Figure 5. The result of this operation is shown in Figure 9. Symmetrization is based on a simple fact that in order to achieve zero velocity, its increase and decrease in the neighboring periods of acceleration and deceleration have to be the same. Specifically, it is performed by checking the areas under the acceleration waveform and comparing the results for adjacent phases. The previously detected ZVS periods are used in order to determine the borders for each acceleration and deceleration phase and so limit the calculated areas. The calculated differences between the adjacent areas enable us to determine an acceleration correction coefficient *a_ACC_*__*COR*_, defined in Equation (5), taking into account also the number of samples in each area.

(5) $$a_{ACC\_COR}=\frac{1}{\left(end-start\right)}\sum_{n=start}^{n=end}a_{SCAL\_ACC\;n}$$

The correction coefficient is then subtracted from or added to each value of acceleration (Figure 9—blue line) to achieve equal areas under the plot in the negative and positive parts of the acceleration waveform where the acceleration and deceleration took place. The final corrected values of acceleration after symmetrization are denoted as *a_ACC_* (Figure 9—red line). However, the symmetrization procedure can be performed only after a certain phase of movement is finished (*ZVS* start and end detected), which results in a delay in this correction. For this reason, the first correction of velocity is necessary and useful, as it can be performed continuously in real time.

**Figure 10:**
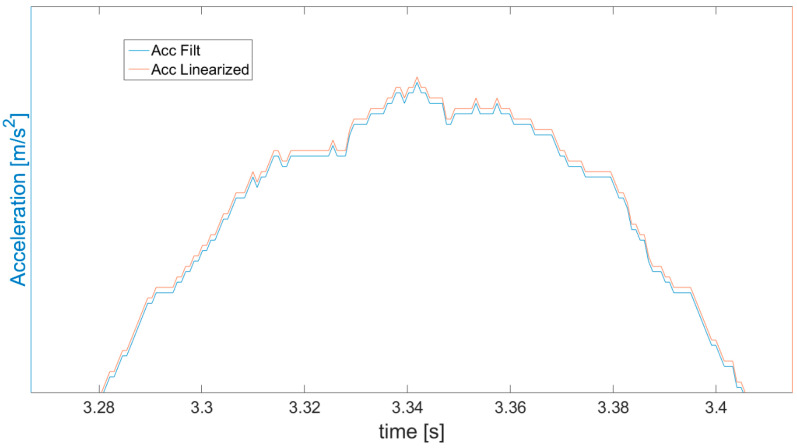
Magnification of part of Figure 9 presenting the difference between accelerations obtained from measurement (blue line) and subjected to symmetrization (red line).

In order to clearly show the symmetrization effect, specifically the difference between the acceleration measured and obtained after symmetrization, part of Figure 9 is magnified and presented in Figure 10. The correction of the acceleration seems to be minor in relation to the scale of the graph, but it has a colossal impact on the further obtained values of velocity and displacement (way), enabling increasing the accuracy and correctness.

**Figure 11:**
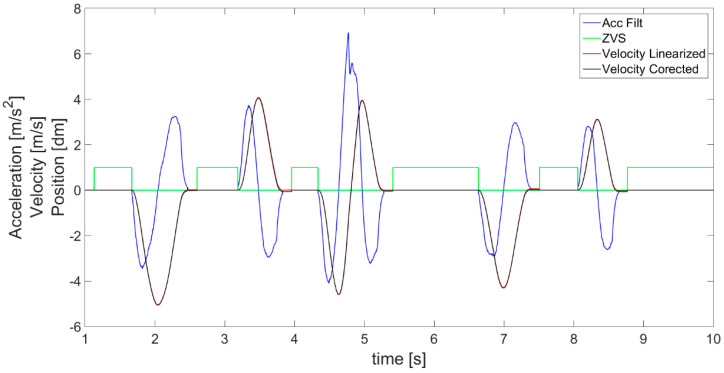
Velocity calculated after acceleration symmetrization (red line—velocity linearized) compared with previous corrected velocity (black line).

The next step is to use the acceleration after symmetrization to calculate the velocity *V_ACC_*, shown in Figure 11 as a red line, according to Equation (6). The obtained result is compared with the velocity achieved with the previous 1st correction (black line). They are mostly consistent with each other. However, even this small difference between them has a huge impact on the obtained displacement. Therefore, symmetrization of acceleration proves to be a much better velocity correction method than the method used previously, subtracting the coefficient determined on the basis of detected ZVS, and after symmetrization, the best effects of estimated velocity are achieved.

(6) $$V_{ACC}=\sum_{n=start}^{n=end}\left(a_{SCAL\_ACC\;n}-a_{ACC\_COR}\right)*dt$$

**Figure 12:**
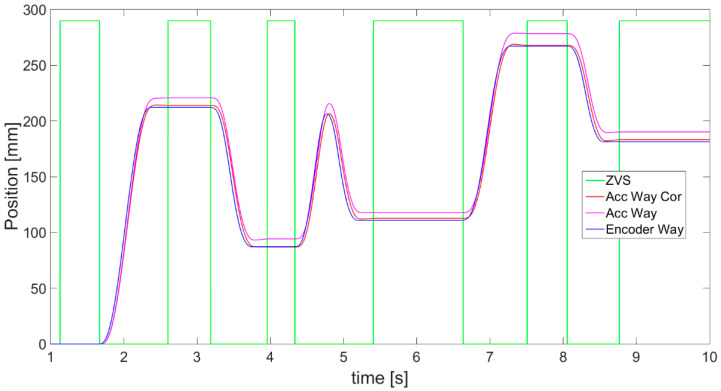
Comparison of displacement (way) measured from encoder (blue), estimated from acceleration after symmetrization (magenta) and calculated from the velocity’s 1st correction (red).

Finally, the accelerometer estimated displacement *S_ACC_* calculated on the basis of final velocity data *V_ACC_*, obtained from symmetrized acceleration, is achieved according to Equation (7) and presented in Figure 12 as a magenta line. In order to evaluate the result, it is compared with displacement obtained from an encoder (blue line). In addition, the red line in Figure 12 refers to the displacement estimated from velocity after first correction, so, based on the velocity indicated with a black line in Figure 11:

(7) $$S_{ACC}=S_{INIT}+\sum_{n=start}^{n=end}V_{ACC\;n}*dt$$

where *S_ACC_* denotes displacement taking place from the measurement starting at *n* = 0, and *S_INIT_* denotes the initial position in case of this research equal to 0.

Displacements obtained from both corrections of velocity are close to the encoder outcomes. However, surprisingly, the results from the 1st velocity correction method (Figure 12, red line) seem to be more consistent with the encoder. On the other hand, what is important is the correctness in stationary parts of movement. This is also more suitable for further intensions of using the accelerometer with OFS and applying sensor fusion. In the red line at about 7.3 s, a small peak is visible, indicating a detected change in the position during a stationary state. It occurred that in other trials, this peak could be much larger, disturbing the whole measurement. This error is not present in the magenta line, indicating the correct displacement based on the symmetrization of acceleration.

### 2.3. Optical Flow Sensor—Displacement Estimation Algorithm

**Figure 13:**
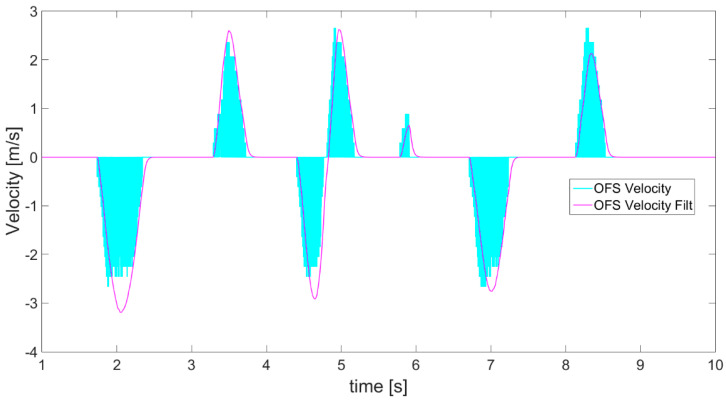
OFS readings of velocity (cyan) and velocity after applying alpha-beta filter (magenta).

**Figure 14:**
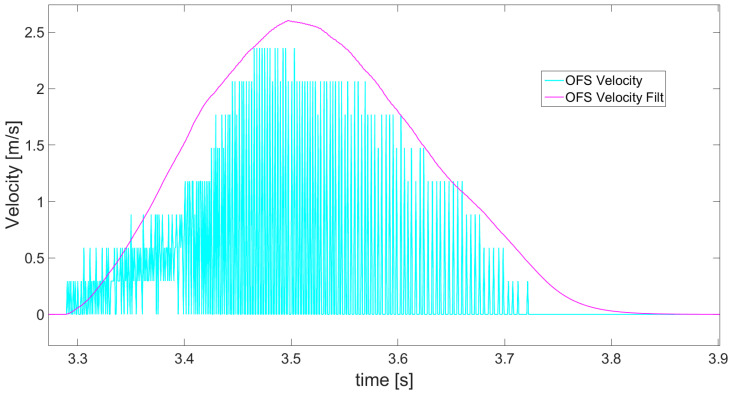
Magnification of part of Figure 13 presenting the noisy OFS readings of velocity (cyan) and magenta line of velocity creating an envelope after applying alpha-beta filter.

The second instrument applied for distance estimation is an optical flow sensor (OFS). Its raw readings regard linear velocity data and, scaled to m/s, these are presented in Figure 13 as cyan line. The optical flow sensor is calibrated by taking one measurement of velocity (also measured by encoder) in both directions, at a fixed distance from the ground. Figure 14 shows even more clearly that the raw data obtained from OFS has a lot of noise and in the velocity waveform seems to produce areas rather than a line. Due to the poor nature of raw OFS readings, an alpha-beta filter, chosen for its simplicity and fast response, is applied to define an envelope, shown as a magenta line in Figure 13 and Figure 14, over the obtained velocity data. Moreover, it is also necessary to correct the sensor’s offset, as its readings are always misleading, implying slightly faster movements in the forward or backward direction. The velocity correction coefficient, depending on direction of movement, is defined experimentally.

**Figure 15:**
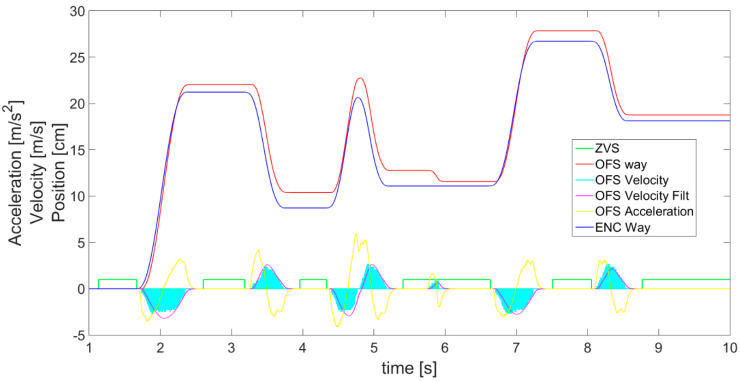
OFS filtered and scaled readings together with those calculated on the basis of the values of acceleration (yellow) and displacement (red).

**Figure 16:**
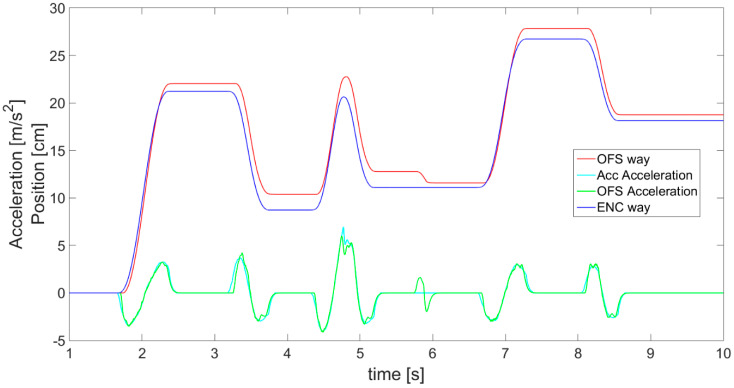
Data for accelerometer and OFS estimated displacements compared with encoder values.

On the basis of filtered and scaled OFS velocity readings, using derivative and integral, the acceleration (Figure 15—yellow), as well as the displacement (red line in Figure 16—OFS) are determined and further compared with displacement measured by the encoder (blue line). In order to obtain smooth acceleration values, again an alpha-beta filter has to be applied on the acceleration data, as with the velocity, to get rid of the noise and stop the third derivative of displacement from being huge. Calculating acceleration is necessary for the next step of sensor fusion where a complementary filter is applied. The delay introduced by alpha-beta filter application was eliminated at the stage of OFS acceleration calculations by shifting the filtered values, which greatly improved the algorithm’s response and final outcomes of complementary filter. The comparison of measured and estimated displacements generally shows a good compatibility between them, proving that the OFS as a sensor and the presented data postprocessing algorithm can both be used for the purpose of displacement estimation.

### 2.4. Sensor Fusion Algorithm—Complementary Filter

**Figure 17:**
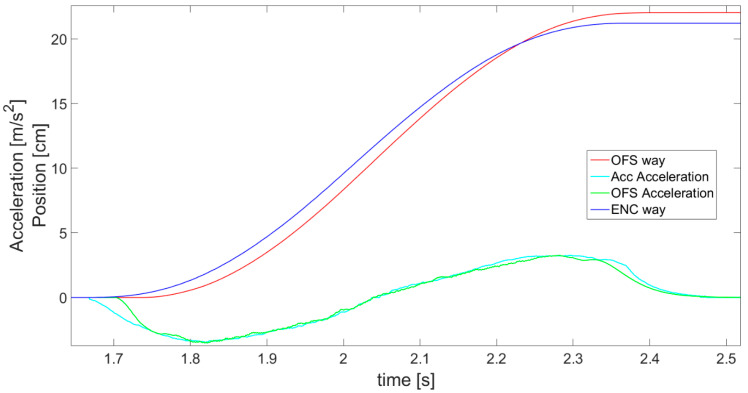
Magnification of part of Figure 16, showing complementary filter operation principles presented with accelerations of both sensors.

After separately studying both sensors, the accelerometer and OFS, a comparison of their results also in relation to encoder’s displacement is presented in Figure 16. The visible fault in the OFS estimated displacement (red line) is the result of an external disturbance, for example an object shifted under the sensor’s lens. This shows that the OFS should be supplemented with readings from another sensor in order to avoid this kind of difficulty. The accelerometer (Figure 12 magenta line) did not detect such a fault, as it does not suffer from this kind of external disturbances. There is also a dissonance in the sensor’s readings visible in Figure 17 in the waveforms of accelerations from the accelerometer and OFS (respectively, the cyan line and green line). This problem may be solved with an application of a complementary filter enabling proper exchangeable or simultaneous usage of data from both sensors and obtaining a correct and reliable displacement estimation.

Due to the above presented reasons, the main idea of this paper is applied and a complementary filter is used to perform sensor fusion of the data from both sensors. The rules standing behind the fusion operation are described below, also presenting the used Equations (8) and (9) applied inside of the complementary filter. The acceleration data provided by the accelerometer and *OFS* are compared in order to determine which sensor’s readings are reliable at a given moment of the measurement and which should be trusted. Specifically for this purpose, a parameter named balance is introduced, and its value is determined according to Equation (8). If both acquired accelerations are small, i.e., lower than a specified limit *a_LIMIT_*, or a *ZVS* is not detected, the balance is acquired as the absolute value of difference between the accelerations from both sensors.

(8) $$Balance=\{\begin{array}{c} \left|a_{\mathrm{SCAL}\_ACC}-a_{OFS}\right|\;for\;\{\begin{array}{c} a_{OFS}\leq a_{LIMIT}\;and\;a_{\mathrm{SCAL}\_\mathrm{ACC}}\leq a_{LIMIT}\\ or\\ ZVS=0 \end{array}\\ \left(a_{OFS}/a_{\mathrm{SCAL}\_ACC}\right)+\left|a_{\mathrm{SCAL}\_ACC}-a_{OFS}\right|\;for\;a_{OFS}\geq a_{\mathrm{SCAL}\_ACC}\;and\;ZVS=1 \end{array}$$

The value of the balance parameter determines the relative importance of velocity data from each sensor and the weights used for the final calculation of velocity estimation *V_Fusion_* according to Equation (9). When balance equals 0, the data for velocity, and in consequence for displacement estimation, come only from the *OFS*, and when balance equals 1, only data from the accelerometer are considered.

(9) $$V_{FUSION}=Balance*V_{ACC}+(1-Balance)*V_{OFS}$$

The work of thre complementary filter is also shown in Figure 17, presenting the aspects on which its operation is based in greater detail. For instance, the difference between accelerations *a_Scal_*__*Acc*_ (cyan line) and *a_OFS_* (green line) can be observed. When this difference is too big, the reliable outcomes are based on the accelerometer, i.e., these will be cases when the accelerometer does not show acceleration and *OFS* does. In addition, a phase shift in the acceleration graphs between sensors is visible as the effect created by the alpha-beta filter. However, this is not considered a problem, since the difference is always the same and it is easily compensated for.

**Figure 18:**
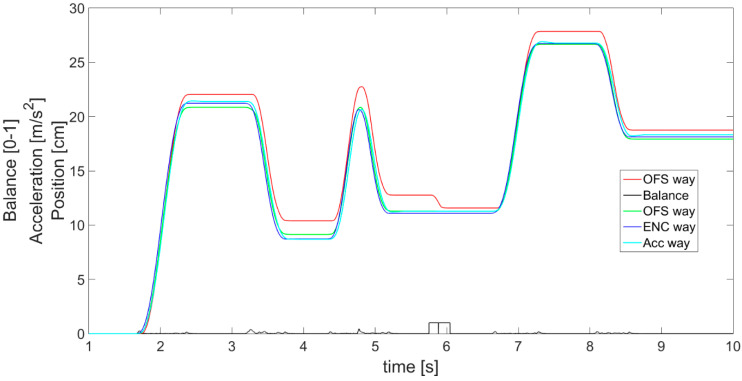
Complementary filter effects presented with displacement plots.

The final values of displacement are denoted as *S_FUSION_* and calculated by performing a derivative of velocity *V_FUSION_*. These results of complementary filter application are presented in Figure 18 as a comparison of displacements, and demonstrate why the implementation of sensor fusion is useful and necessary. In Figure 18, the displacement *S_FUSION_* is indicated with a green line, whereas a black line shows the values of balance.

**Figure 19:**
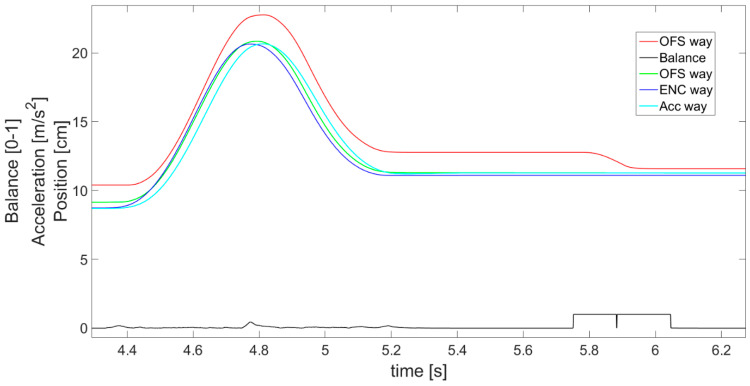
Magnified view of part of Figure 18, presenting a close comparison of displacements.

Furthermore, it is visible in Figure 19 that the complementary filter is good at finding errors from the optical flow sensor. The OFS-estimated displacement (red line) is not concise with the reference encoder data (blue) and, for instance, at about 5 to 6 s, the OFS error is evident. Meanwhile, the displacement estimated from the complementary filter (green) is kept close to the encoder values even when OFS provides data with an error. In addition, the complementary filter allows earlier detection of velocity change, realized by the accelerometer, than just by using OFS. This, as a result, reduces the error in the initial part of the acceleration phase and in the final part of the deceleration phase, when the actual velocity is too low for the OFS sensor to detect it.

It should be emphasized that in Figure 18 and Figure 19, the displacement estimated on the basis of the accelerometer (cyan line) seems to achieve the desired precision and stability. However, in reality, if the object moves at a constant speed for a long time, it is necessary to use the reading from the OFS (red line). This approach gives much better results than the accelerometer in this kind of movement. On the contrary, when the speed changes rapidly, the accelerometer is much better. This is the basis for explaining the need for complementary filtration and fusion of the two sensors. Overall, the figures clearly present the differences between the obtained results and prove that the applied sensor fusion is useful, appropriate, and meaningful.

## 3. Results

**Figure 20:**
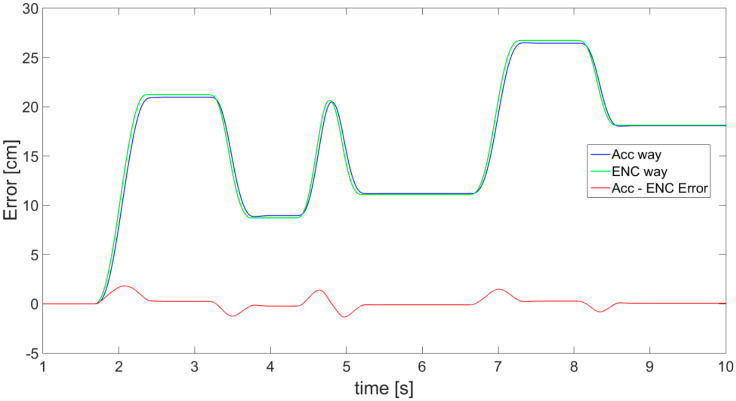
Results of displacement: estimated with accelerometer (blue), measured with encoder (green), and calculated difference between them (red).

**Figure 21:**
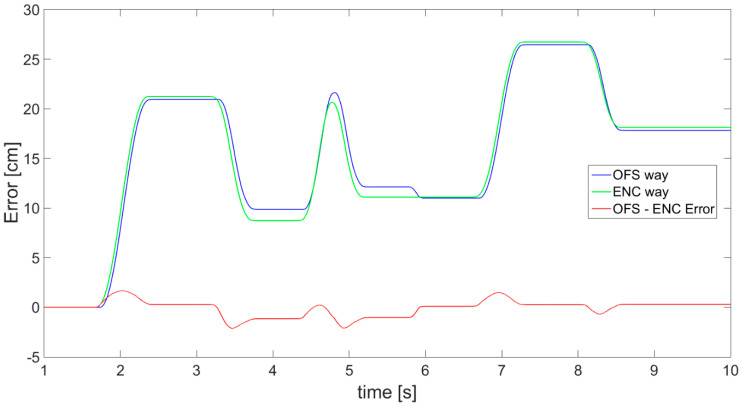
Results of displacement: estimated with OFS (blue), measured with encoder (green), and calculated difference between them (red).

**Figure 22:**
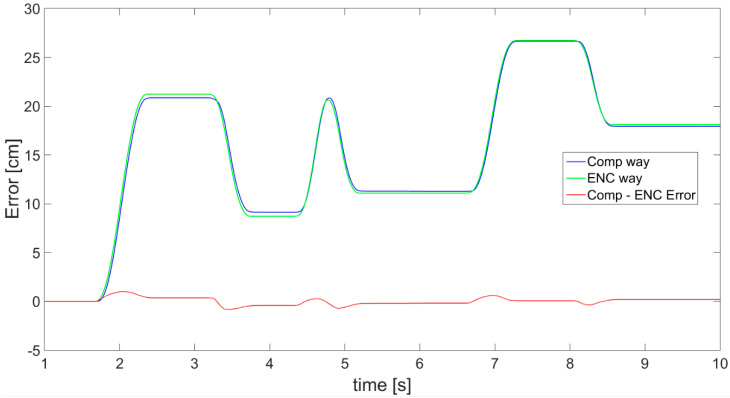
Results of displacement: estimated from sensor fusion between the accelerometer and OFS with usage of a complementary filter (blue), measured with encoder (green), and calculated difference of displacements (red).

**Figure 23:**
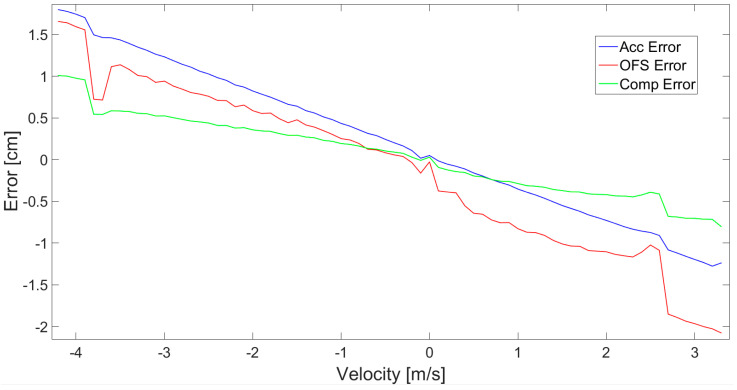
The displacement error in relation to movement velocity for 3 experimental cases.

After the data postprocessing algorithms for each sensor and complementary filter were determined, they were applied to conduct an experiment using the earlier described test stand. The experiment followed the same procedure as during data acquisition presented in Section 2. A planned linear movement of the platform with both sensors was enforced and the level of accuracy of the displacement estimation was evaluated. In addition, an external disturbance was again introduced in the form of a flat object placed under the linear guide in order to measure and evaluate the OFS results in such a case. A serial interface was used to send the obtained data from accelerometer and encoder to a computer. Detailed outcomes of experiment are presented as waveforms in Figure 20, Figure 21, Figure 22 and Figure 23. Comparison of displacement results measured with an encoder and estimated with the usage of both sensors was carried out. The values of error calculated by subtracting the obtained displacements are also presented.

In Figure 20, outcomes of the displacement estimation, based on an accelerometer (blue), and the application of the developed algorithm described in Section 2.2, are presented. Displacement measured by the encoder is presented with a green line, whereas the red line shows the error between displacements.

Similar results achieved with application of OFS are shown in Figure 21. It must be emphasized that a fault, in comparison with encoder data (green), is visible, indicating the existence of detection error and resulting in the inaccuracy of OFS displacement estimation (blue). This is the result of an external disturbance, also clearly visible in the error line (red).

Finally, the complementary filter application effects are presented in Figure 22, showing very promising results, since the error (red) is small. In addition, for all three cases, the dependency of the displacement estimation error value in relation to velocity of movement is presented in Figure 23.

Figure 20, Figure 21, Figure 22 and Figure 23 all show a relatively small error of displacement estimation, but Figure 22 and Figure 23 prove that the best results are obtained by performing data fusion with a complementary filter.

The numerical data of distance estimation errors, calculated separately for the accelerometer, OFS, and fusion with a complementary filter, are summarized in Table 1. Max error is the absolute maximum value of error deviation from zero. AVG error is the average of deviation from 0. The values of variance error are calculated as the variance of the measurement error waveform, where the expected value is 0.

Generally, the numerical data of error (Table 1) obtained for accelerometer and OFS are comparable. The maximum value of error for accelerometer values is about 2.6 cm, and is higher than for the OFS, at about 2.1 cm. Meanwhile, the average and variance are smaller in the case of the accelerometer. However, the best results are obtained in the case of the complementary filter application. All the numerical values characterizing error are much smaller than in case of the two sensors used separately. For the fusion case, the maximum error is just slightly larger than 1 cm, average values are about 0.03 cm, and the variance error does not exceed 0.13 cm. This further proves that the data sensor fusion works fine and the obtained precision is satisfying, since the error values are minor.

## 4. Discussion

There is an increasing need for an easy, cheap, and most of all accurate and reliable localization system and methodology. This is true not only in the case of mobile robots and the growing market of indoor services and their application for outdoor tasks, but also personal localization, for instance the in case of pedestrians. In many solutions for displacement estimation, the usage of various inertial navigation systems is considered [16,20,22]. Mobile robots’ odometry is often calculated by optical flow sensors, as in [5,10,13], where inertial sensors are also used, along with the OFS. However, in these cases, they are applied to obtain the angular orientation data or evaluate the results, as for instance in [12], where OFS is supplemented with a gyroscope. Regarding our goal, our research is similar to the concept presented in the above articles and in [14], where displacement estimation was based purely on OFS, while the IMU sensor was still used to obtain orientation data.

However, in this paper, the new idea is to apply an inertial sensor (accelerometer) as a secondary source of displacement estimation, and in this way, with the usage of a developed complementary filter, achieve sensor fusion and directly supplement the output data of the OFS, improving the accuracy and reliability. Hence, the paper’s idea is consistent with the cited articles, but to a certain degree, and only in conclusions of [5] was a similar idea of using sensor fusion of OSF and an inertial sensor to overcome the possible OSF problems introduced. Furthermore, this article also intends to improve the ZV detection algorithm to avoid still-phase leakage detection and over-detection, as mentioned in [16], where gait was measured. The detected zero velocity states are then used to perform the first correction of velocity and the acceleration symmetrization (linearization). This novel approach allowed us to achieve the best observed values of velocity and, in consequence, distance estimation.

As presented in this paper, the emphasis was placed on the application of inertial and optical flow sensors for determining a platform’s movement on a smooth surface. The platform’s concept may be associated with an automated guided vehicle (AGV) robot or a computer mouse. For this purpose, a motor-powered test stand with a linear guide was developed and built. Algorithms for distance estimation from an accelerometer and OSF were implemented and tested individually. Above all, sensor fusion of both sensors was applied as a complementary filter of accelerations, for the purpose of improving the accuracy of displacement estimation procedure.

Observation and analysis of results of each step of the accelerometer algorithm proved that it works perfectly fine. Of great importance is the implementation of velocity correction method depending on the detection of stationary state and the symmetrized acceleration, which greatly enhanced the effects of the displacement estimation. The improvement is so significant that it seems that the accelerometer could also be used independently for distance estimation. However, its readings may become less precise with time, due to its accumulating errors from vibrations. Furthermore, estimation of distance in cases of movement with constant velocity poses a challenging problem. For such cases, OFS is more precise and reliable, but this sensor also has a couple of drawbacks and may be distorted. For this reason, using OFS in combination with another sensor is a good way to supplement its limitations, compensate for its errors, and help to overcome these possible difficulties. The goal was to determine, as simply as possible, the moments in which the indications of one of the sensors were so trustworthy and accurate to become an input data vector for the temporary “automatic tuning” of the further part of the algorithm’s work. These states were defined as zero velocity states. It would also be possible to use a recursively minimized object state vector variance. However, this method, called the Kalman filter, does not allow a 100% sudden isolation of the algorithm from the sensors’ temporarily disturbed data. Hence, it was decided to implement some of the algorithm’s operation in the form of rigid rules defined during the observation of each sensor’s separate errors. This approach to the problem appeared to be sufficient to detect when the accelerometer is wrong and when the OFS is wrong. The system receives not only the signal from both sensors with white measuring noise, but also indirectly information about when to trust each sensor.

Another goal of the fusion algorithm is to determine the object’s velocity not only in ZV states, but rather to eliminate the velocity drift problems, which occur at the double integration of accelerometer data, on the basis of OFS just supplemented with an accelerometer. For this reason, the developed algorithm with a complementary filter is no longer a ZVS or ZUPT algorithm, but rather may be called a true velocity state algorithm (TVS). Additionally, an important aspect of the fusion is the ability to detect and compensate for possible errors of the OFS-based vision system that may arise due to shifting/encountering an accidental physical object under its lens (external disturbance).

The study of the platform’s estimated displacement and its deviation from the encoder outcomes was used as the method to evaluate the efficiency of individual algorithms and improvements when fusion was applied. Obtained results and errors can be indirectly compared with outcomes presented in [25], where translations were estimated with a three-axis accelerometer placed on a shaking table. The found displacements shown in the figures indicate that algorithms are applicable for the displacement sensor to track moving objects with good precision. A small drawback of the applied fusion algorithm is visible in Figure 18, which is a delay in calculated displacement, present due to the application of acceleration symmetrization. However, this is not a problem since the first velocity correction is performed in real time, meaning that a continuous flow of corrected displacement is obtained and the correction resulting from symmetrization is executed from time to time compensating for accumulating errors. The results are repeatable within the error limits and resistant to noise amplitude changes resulting from the movement of the object on the ground, providing that the measuring noise does not exceed 2–5% of the sensor’s measuring range. When the object is moving, the noise always exists, and it is already a signal for the system regarding non-zero velocity detection. While the flatness deviation is maintained, the system remains stable. However, the system will not be stable when the angular orientation of the object with respect to the Earth’s gravitational field by changes more than 15’, i.e., when the gravity vector’s projection on the accelerometer measuring axes changes. For angular orientation deviations larger than 15’, the error is larger than 3 bits read by the analog to digital converter. This drawback will be eliminated in future versions of the algorithm being developed. The algorithm is also not resistant to accelerations with too fast amplitude changes, i.e., too high frequency of changes. This is the result of a specific and constant maximum sampling rate of the accelerometer at 1100 Hz. All in all, the outcomes of the experiment suggest that the fusion of sensor data is correct and useful. Therefore, the final aim of this research is achieved and a system that is able to estimate the moving object’s linear displacement in relation to the ground using just the OFS and accelerometer is obtained.

Future planned improvements of the system include applying fusion for even more sensors. The idea is also to use a self-adaptable lens for the OFS and a distance measuring sensor. This would enable adjusting the focal length in order to obtain the best possible result of measurement in the case of varying height of the OFS placement above the ground.

## Figures and Tables

**Table 1:** Numerical data of distance estimation error calculated for each experimental case separately.

Experimental Case	Max Error [cm]	AVG Error [cm]	Variance Error [cm]
Accelerometer	2.61	0.1165	0.3245
OFS	2.132	-0.812	0.6448
Fusion	1.0142	0.0331	0.1266

## Data Availability

The data presented in this study are available on request from the corresponding author. The data are not publicly available due to technical reasons and planned application in industry.
